# New Perspectives on Ancient Granulomas

**DOI:** 10.3389/fimmu.2013.00345

**Published:** 2013-11-15

**Authors:** Dov L. Boros

**Affiliations:** ^1^Department of Immunology and Microbiology, Wayne State University, School of Medicine, Detroit, MI, USA

**Keywords:** pathogens, inflammation, granuloma, pathogen sequestration, pathogen protection, granuloma necrosis, pathogen dispersal

Granulomas (tubercles) were described by pathologists as the hallmark of tuberculosis (TB), an ancient, often lethal disease. The discovery by Koch of the bacilli [*Mycobacterium tuberculosis*, (M.tb)] as the causative agent of the disease initiated research that established the paradigm of the protective but also tissue-destructive nature of the granulomas. These Reviews provide an update on research in granulomatous infections/diseases and describe a shift into a new paradigm: the role of the pathogen in host-pathogen interaction.

The protective nature of the granuloma is based on phagocytosis and killing of the ingested pathogen driven by the innate and adaptive immune responses. On bacterial invasion the antigen presenting cells, macrophages via their pattern recognition receptors recognize the pathogen associated molecular patterns of the pathogen and initiate the defensive inflammatory response. Itoh et al. (1) present evidence that in the Bead-PPD lung granuloma model the innate TLR9 and the Dll4 Notch systems cooperate not only in the early recognition of M.tb patterns but also in skewing the incipient adaptive immune response toward the Th17 inflammatory cell activity. Granuloma formation is reliant on chemokines, the low MW proteins that recruit leukocytes to the focus of irritation and activate them. In Stephen W. Chensue’s (2) laboratory the artificial granuloma model was established by means of antigen coated beads injected into Th1 or Th2 response-primed mice. The resulting lung granulomas showed the TH1 or Th 2 type cellular response. Chemokine receptor analysis of such granulomas showed lesion age-dependent heterogeneity of the response. It is suggested that chemokine’s could be targeted for therapeutic intervention.

Ehlers and Schaible (3) give an overview of the M.tb granuloma research and the host-pathogen interaction. The protective role in TB of the granuloma is asserted, but reflecting the recent view M.tb is described to exploit the lesion for its dispersal and propagation. At the chronic stage of the disease bacilli within the lesion are dormant and adapt to low oxygen, pH, and nutritional conditions. The pathogen-induced strong TH1 type inflammation causes pathology: caseation, cavitation that allow the escape of the bacilli to infect a new host. The authors make the intriguing suggestion to focus on suppression of the intensity of inflammation rather than devising new vaccination modes that maintain the inflammatory response. Shaler et al. (4) describe the stages of the M.tb granuloma development with the appearance within the lesions of the non-phagocytic macrophage derivatives: foam, epithelioid, and giant cells. They take the negative view of the lesions because they contribute to bacillary persistence and based on the newly emerging zebra fish embryo model contend that granulomas may be dispensable. Lang (5) discusses the relevance of Mincle a C type lectin on macrophages that is the receptor for bacterial cord factor – a toxic glycolipid that incorporated into adjuvant induces granulomas. Linkage with TLRs could not be demonstrated and so far experiments with Mincle-deficient mice showed discrepant results related to anti-bacterial resistance. Lugo-Villarino et al. (6) give an account on the importance of the M1 M2 polarization that occurs during M.tb infection. Under the early TH1 (IFNg) influence M1 macrophages become strongly bactericidal. This shifts to a late Th2 (IL4, 10) M2 response with Arginase 1 expression that inhibits nitric oxide production resulting in poor or weak bactericidal capacity and the appearance of foamy macrophages that allow intracellular bacillary growth. Guirado and Schlesinger (7) confirm the protective role of the Th1-induced M.tb granuloma with strong M1 mediated bactericidal kill. They rightly stress the importance of the microenvironment in and around the granulomas that cause a variety of pulmonary pathologies in a single person and they acknowledge the changing dynamics between host and pathogen during the long course of the infection. They enumerate the various *in vivo* TB models, the *in vitro* efforts and the emergent *in silico* approaches whereby the complex granuloma response is being simplified by computer simulation.

Moore et al. (8) introduce visceral leishmaniasis a chronic tropical disease caused by the protozoan *Leishmania donovani*. In humans or mice host resistance is linked to efficient granuloma development. The murine model has several different characteristics. After infection the parasites are ingested by liver Kupffer cells which together with invariant natural killer T cells recruit monocytes. Both infiltrated CD4+ and CD8+ T cells are needed for parasite clearance. By 4D intravital imaging free movement of intralesional T cells was observed. Parasite clearance proceeds with no residual pathology (necrosis, fibrosis). The innovative computer modeling is being employed to simulate the effector cell populations and predict regulator cytokine activity.

Lundy and Lukacs (9) review the helminth-induced granulomatous schistosomiasis *mansoni*, where antigens secreted by the parasite eggs induce perioval granulomas that protect the liver from toxic egg secretions. The initial Th1 type inflammation, with chronicity is modulated to a Th2 response that significantly reduces granuloma size but enhances liver fibrosis. Downmodulation is mediated by T regulatory and CD5+ B cells that induce apoptosis of CD4+ T cells. It is also systemic for unrelated immune systems which has implications for vaccinations, allergy, and autoimmunity. Hams et al. (10) discuss the host parasite relationship in murine schistosomiasis. The parasite needs perioval granuloma formation for the successful egg excretion from the host. Host granulomas shield the liver from toxic egg secretions. But the predominant Th2 response generates eosinophile rich granulomas and excessive fibrous healing that cause portal hypertension and intestinal bleedings. CD8+ T cells, B cells, and M2 type macrophages play a role in granuloma regulation. Egg antigens are also active at the innate response level: they suppress TLR triggered TNFalpha production by dendritic cells but activate the NLRP3 inflammasome with IL1beta production. Proteomic analysis of egg antigens revealed over 1000 proteins indicating the daunting task of assigning roles to fractions in inflammation or pathology.

The compiled Reviews show significant advances in granuloma research both in new methodologies and the emergence of new paradigm(s) in host-pathogen interaction. In the various models several contributors rightly reaffirm the essential protective role of the immune granulomatous response. However, in TB the quandary of the strong T cell-mediated anti-bacterial inflammation vs. the tissue-destructive pathological sequelae still remains unsolved. Similarly, in schistosomiasis there is no intervention that could prevent or reverse the strong fibrosis that replaces the perioval granuloma.

It is instructive to compare the various experimental models with pulmonary sarcoidosis, a granulomatous disease of humans with no known etiologic agent(s). Broos et al. (in review) survey the current literature that proves the immune basis of the disease. Clinical observations described innate responses with cytokine/chemokine secretion, macrophage recruitment, and inflammatory Th1/Th17 T helper lymphocyte participation in the granulomatous response. The disease may spontaneously resolve or may progress to fibrosis, lung pathology and death. Regulatory T cells (Treg) are considered to restrain overt T helper cell activity and the extent of inflammation but if they are dysfunctional, disease progression occurs. Thus, the clinical picture closely resembles the experimental animal models. Further observations are needed to unravel Th-Treg interaction and the precise role of Treg before effective therapy can be devised.
